# Multiple Therapy Approach for Stage 3 Coats Disease: Long-Term Follow-Up

**DOI:** 10.1155/2020/8840630

**Published:** 2020-10-26

**Authors:** Rodolfo Mastropasqua, Rossella D'Aloisio, Paulo Eduardo Stanga, Richard Haynes

**Affiliations:** ^1^Bristol Eye Hospital, Bristol, UK; ^2^Institute of Ophthalmology, University of Modena and Reggio Emilia, Modena 41121, Italy; ^3^Ophthalmology Clinic, Department of Medicine and Science of Ageing, University G. D'Annunzio Chieti-Pescara, Chieti 66100, Italy; ^4^School of Biological Sciences, Faculty of Biology, Medicine and Health, University of Manchester, Manchester, UK

## Abstract

**Purpose:**

To assess long-term efficacy of a multiple therapy approach in the treatment and management of unilateral stage 3 Coats disease with exudative retinal detachment.

**Methods:**

2 eyes of 2 young patients suffering from unilateral stage 3 Coats disease underwent a multiple therapy approach consisting of surgical drainage of exudative subretinal fluid + one simultaneous and up to one subsequent intravitreal injection of bevacizumab + multiple (up to 3) laser photocoagulation sessions of retinal nonperfusion areas and leaking Coats vasculature.

**Results:**

Complete reabsorption of SRF and retinal reattachment were observed in both cases over the follow-up. In no cases, we observed progression to phthisis bulbi. No bevacizumab-related complications were observed. Epiretinal membrane onset was detected in one eye at the end of follow-up.

**Conclusion:**

The management and treatment of this rare and degenerative disease in young subjects are still a challenge. The described technique is less invasive than conventional intraocular surgery and may be preferable to halt the devastating progression of the disease.

## 1. Introduction

Coats disease remains a progressive and devastating retinal vascular disorder with an estimated population incidence in the United Kingdom of 0.09 per 100,000 people, mainly in young males aged from 8 to 16 years, although adult-onset has been rarely reported [1, 2].

In 2001, Shields et al. proposed a detailed classification for this rare disease based on clinical features: stage 1 (retinal telangiectasia only), stage 2A (telangiectasia and extrafoveal exudation), stage 2B (telangiectasia and foveal exudation), stage 3A1 (subtotal extrafoveal exudative retinal detachment), stage 3A2 (subtotal exudative retinal detachment involving the fovea), stage 3B (total exudative retinal detachment), stage 4 (total exudative retinal detachment with secondary glaucoma), or stage 5 (end-stage disease, phthisis bulbi) [3].

Unfortunately, the exact pathogenesis of Coats disease is unknown, and its diagnosis is often delayed due to its great variability in appearance. Several treatment options based on stages have been proposed: for stages 1 and 5, observation; for stage 2, retinal laser photocoagulation as a first-line; and for stages 3 and 4, many options described starting from sclerotomy and drainage of subretinal fluid, buckling or encirclement, vitrectomy with gas or silicone oil tamponade [4].

A new treatment approach for stage 3 Coats disease was described by Stanga et al. [5], combining three different procedures such as transscleral drainage of SRF, intravitreal anti-VEGF injection, and retinal nonperfusion area laser photocoagulation. They reported their successful experience in 8 eyes of children with a variable follow-up from 9 to 60 months.

The aim of our case series was to assess long-term efficacy of a multiple therapy approach in the treatment and management of unilateral stage 3 childhood-onset Coats disease with exudative retinal detachment.

## 2. Methods

### 2.1. Study Participants

In this retrospective interventional case series, 2 eyes of 2 children suffering from unilateral stage 3 Coats disease received a successful multiple therapy approach as described below.

The affected eyes were classified according to Shields' classification as stage 3B (Case 1: total exudative retinal detachment) and stage 3A1 (Case 2: subtotal extrafoveal exudative retinal detachment, not involving the fovea).

Inclusion criteria were as follows: (i) children with a diagnosis of unilateral Coats (stage 3, according to Shields' classification) and (ii) no history of previous ocular surgery or other treatments including laser or cryotherapy.

Exclusion criteria were as follows: (i) evidence or history of other ocular conditions; (ii) evidence or history of systemic disorders; and (iii) other stages of Coats disease.

For both cases, a complete ophthalmological evaluation, best-corrected visual acuity (BCVA) assessment, and OCT were performed before surgical procedure and at all follow-up visits.

### 2.2. Surgical Procedure

In one case (Case 1), a triple therapy approach was performed as previously described by Stanga et al [5]: under general anesthesia, exudative subretinal fluid was drained transclerally by a 27-gauge needle mounted on a 5 ml syringe with the plunger removed. In order to avoid retinal damage, the needle was inserted perpendicularly to the sclera in the area having the higher amount of fluid (previously identified with binocular indirect ophthalmoscopy). An anterior chamber maintainer was used to keep an adequate eye pressure during the drainage. Thereafter, an intravitreal injection of bevacizumab and laser photocoagulation of retinal nonperfusion areas and leaking Coats vasculature were performed. Another bevacizumab injection and 2 more sessions of laser photocoagulation were performed during the follow-up.

In the other case (Case 2), no drainage procedure was performed because of the exudative retinal elevation appeared to be a retinoschisis as described below in detail. Both patients received injection of 1.25 mg intravitreal Avastin® (30 G needle) combined with binocular indirect argon laser photocoagulation of microaneurysms, telangiectatic vascular changes, and areas of nonperfusion. Imaging wide-field fundus fluorescein angiography treatment guidance Optos® was used in the two eyes. No intraoperative complications were observed.

## 3. Results

**Figure 1:**
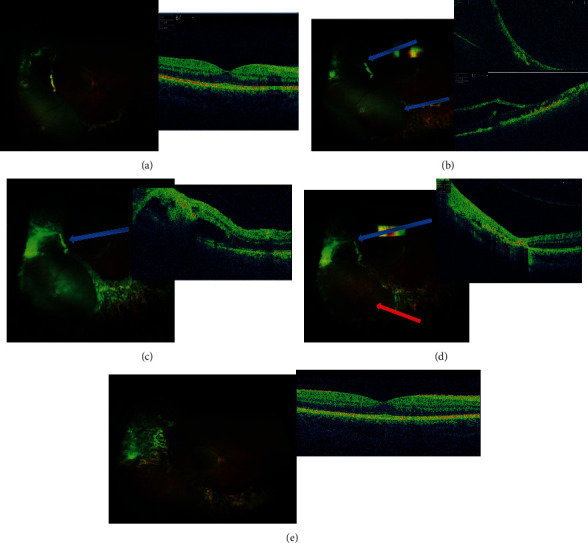
Case presentation of a 16-year-old patient treated with multiple therapy approach for Coats disease. (a) At presentation, the patient had a stage 3A Coats disease with the macula spared from exudation (VA 0.10 LogMAR). (b) 1 month after surgical treatment: note the laser scars and the persistence of SRF temporally, whereas the inferotemporal area appears at the OCT as a retinoschisis (arrows). The patient underwent a second treatment. (c) 1 week after the second session of laser. *Note.* The fresh scarring tissue and the gradual reabsorption of SRF at the OCT (arrow). The patient received a third treatment. (d) 1 month after the last treatment. *Note.* The total reabsorption of SRF (blue arrow). The red arrow shows the presence of laser scars, which confirms the diagnosis of retinoschisis. (e) 8 months after the first treatment. The SRF is completely reabsorbed and the clinical picture is stable. *Note.* The development of a mild and asymptomatic ERM at the OCT (VA 0.0 LogMAR).

A total of 2 eyes of 2 children aged between 16 and 17 and affected by unilateral stage 3 Coats disease were considered in the study. Case 1 required only 1 episode of drainage of SRF. The second case (Case 2) with significant elevation of the retina did not require drainage since the exudative retinal elevation appeared to be a retinoschisis rather than a full-thickness exudative retinal detachment. This became apparent during laser treatment when laser burns applied to the bed of the elevated retina showed “whitening of the outer leaf,” indicating a thin layer of the retina was still in contact with the RPE and was, therefore, a schisis (Figure 1).

Demographic and clinical features of the two patients enrolled are reported in Table 1.

Family history was negative in both cases.

Reabsorption of SRF and total retina reattachment were observed in all two cases during the follow-up (Table 1 and Figure 1).

In Case 1, BCVA remained stable (light perception) after surgery and for whole follow-up without recurrence of SRF and retinal detachment (Table 1). In Case 2, a significant improvement in terms of BCVA was assessed (from 0.10 LogMAR to 0.0 LogMAR), even though an initial epiretinal membrane onset was found at the end of follow-up.

No bevacizumab-related complications were observed. No further disease progression was observed in all cases.

## 4. Discussion

Coats disease is a sporadic, nonhereditary retinal vascular disorder mainly affecting young males. Adulthood-onset disease is very rare and has been described as less aggressive with a slow development and a more favorable treatment outcome [2].

A definitive gold standard treatment for Coats disease has not been established yet [3].

Indeed, the management and treatment of such a rare and degenerative disease in young subjects are still a challenge. Ideally, the least invasive approach would be preferable to halt the devastating progression of the disease. The choice of follow-up and treatment depends on disease staging [6]. In spite of treatment, many advanced Coats disease-related complications have been reported in previous studies, such as rubeosis iridis, neovascular glaucoma, phthisis bulbi, and ocular pain leading to enucleation [7].

The external SRF drainage has previously been described to successfully prevent neovascular glaucoma onset [8].

Intravitreal anti-VEGF injections have showed a temporary efficacy in the reduction of capillary leakage and the consequent exudation that eventually leads to exudative retinal detachment [9–11]. Retinal laser photocoagulation has been reported to be effective in ablating the abnormal peripheral retinal vasculature that characterizes this pathology [12].

Other more invasive surgical procedures have been performed such as pars plana vitrectomy with silicone oil or gas tamponade or scleral buckling in order to solve the exudative retinal detachment and to halt progression of advanced-stage Coats disease [13].

In 2016, Stanga et al. [5] were the first to propose a new treatment multiple approach for stage 3 Coats disease combining three different procedures: transscleral drainage of SRF, intravitreal anti-VEGF injections, and laser photocoagulation of the nonperfused area of the retina.

Similarly, our work reported anatomical and functional outcomes of 2 eyes with a diagnosis of unilateral stage 3 Coats disease: one case was treated with the triple approach consisting of external SRF drainage, consecutive bevacizumab intravitreal injections, and multiple wide-field image-guided photocoagulation laser sessions and the other case was treated with laser + anti-VEGF treatments alone because of the schisis.

In the case with total exudative retinal detachment (Case 1), the SRF drainage procedure was essential to allow apposition of the retina against the RPE in order to allow uptake of the laser by the detached retina. When the SRF is sufficiently drained, scleral indentation during the indirect argon laser treatment allows more complete laser photocoagulation of the ischemic areas and telangiectatic vessels. Care must be taken during lasering to not allow too much heat to accumulate in one spot; otherwise, the resulting “pop” could potentially inadvertently convert the exudative detachment into a rhegmatogenous detachment. The intravitreal anti-VEGF injection is designed to halt the ongoing leakage from retinal vessels while the laser takes effect over the subsequent weeks. Further laser treatment is usually carried out after the first session as the anti-VEGF will temporarily dry the subretinal space, further allowing better uptake of a laser at the second session. If sufficient photocoagulation of the leaking areas has occurred, this prevents recurrence of leakage and subretinal fluid accumulation when the anti-VEGF injection wears off. The indirect laser technique may need to be modified with the application of longer duration burns in order to achieve uptake and complete coagulation of the abnormal vasculature. As mentioned above, care must be taken to avoid too much laser energy being concentrated at one spot. Our results showed an excellent anatomical outcome with a total reabsorption of SRF and a complete retinal reattachment without relapses during long-term follow-up. Of note, a second case (Case 2) showed an excellent BCVA (0.0 LogMAR), which was maintained over the follow-up. This is probably related to the staging (3A) differently from the other case enrolled who was classified as more advanced (3B). Interestingly, this second case had an exudative retinoschisis rather than a full-thickness retinal detachment and developed an ERM during the follow-up as ate complication, possibly due to the multiple sessions of laser treatment and/or breakdown in the blood-retinal barrier secondary to the primary pathology. Despite the ERM, we observed an excellent BCVA restoration presumably because it was early without a significant traction. This positive visual outcome was quite unexpected but very welcoming when considering the usual visual outcomes of patients affected by advanced Coats.

As reported by Shields in their classification of 2001, 74% of patients with stage 3 Coats have a poor visual outcome of 20/200 or worse. Moreover, they state that visual acuity goes parallel with staging of patients [1].

Shields et al. thoroughly described possible predictors of enucleation that represent often the final step of Coats management failure, considering age category and degree of subretinal fluid [1]. A younger age is strongly associated with a worse disease presentation and final functional outcome [1].

In our two cases, similarly to Stanga et al., a multiple therapy approach including at least 2 consecutive intravitreal injections of anti-VEGF was performed. The role of VEGF in the pathogenetic mechanisms of Coats disease, particularly in vascular abnormalities and capillary exudation, is already known. The two main factors that have a key role in Coats pathogenesis are retinal vessel endothelial changes responsible for the breakdown of blood-retinal barrier and the presence of abnormal pericytes causing telangiectatic vessels, with the typical appearance of “light bulb telangiectasias,” due to the terminal shape of peripheral vasculature and yellow exudation [2].

Nevertheless, much caution should be taken when using anti-VEGF due to the possible risk of fibrosis and consequent tractional retinal detachment [5].

In their retrospective case note review, Stanga et al. [5] reported that this new therapeutic and less invasive multiple approach was safe and long-term effective in 8 eyes of children, avoiding the need for vitrectomy. Unfortunately, 3 of them developed cataract as a late complication. We did not observe any complication, apart from 1 case of early epiretinal membrane that is not yet significantly affecting the visual outcome. A strict follow-up is necessary to evaluate ERM development. Our work has some limitations such as the small case series of children considered, the relatively short follow-up, and the lack of a genetic workup, even if Coats disease is sporadic and nonhereditary in its strict definition.

Obviously, further cases and a wider follow-up are required to make these data consistent. It should be remembered that many patients with Coats disease are often undiagnosed until advanced stages. A prompt diagnosis, an accurate management, and treatment of such a rare disease affecting very young subjects are still challenging.

Other retinal pathologies can simulate an “exudative retinopathy” including branch retinal occlusion, ocular toxoplasmosis, familiar exudative retinopathy, retinal capillary hemangiomatosis, etc., confounding the final diagnosis [3].

A less invasive approach and a more conservative staged treatment regime, particularly in childhood-onset Coats, may be preferable to halt the devastating progression of the disease, aiming to minimize the possible recurrence of exudation and subretinal fluid formation.

In conclusion, our case series have shown promising results in controlling and stabilizing this blinding condition, confirming the previous findings presented by Stanga et al. in terms of safety and long-term efficacy of SRF drainage, followed by anti-VEGF intravitreal injections and laser photocoagulation in stage 3 Coats disease and without SRF drainage in stage 3 Coats associated with schisis.

## Figures and Tables

**Table 1:** Demographic and clinical features of the patients enrolled.

Case	Eye	Age	Stage B	SRF drainage	No. of bevacizumab injections	No. of laser sessions	F up	Preop VA	Postop VA	Complications
1	RE	17	3	Y	2	3	35	LP	LP	None
2	RE	16	3	N	3	3	22	0.10	0.0	ERM

Preop: preoperative; postop: postoperative; VA: visual acuity (expressed in LogMAR scale); F up: follow-up; SRF: subretinal fluid; Y: yes; N: no; LP: light perception; RE: right eye; ERM: epiretinal membrane.

## Data Availability

The data are available upon request from the corresponding author.

## Additional Points

*Summary*. A multiple therapy approach in unilateral stage 3 Coats disease is less invasive and very effective in long-term follow-up.
